# Increased breast cancer mortality only in the lower education group: age-period-cohort effect in breast cancer mortality by educational level in South Korea, 1983-2012

**DOI:** 10.1186/s12939-017-0554-6

**Published:** 2017-03-31

**Authors:** Jinwook Bahk, Sung-Mi Jang, Kyunghee Jung-Choi

**Affiliations:** 1 Department of Public Health, Keimyung University, 1095 Dalgubeol-Daero, Dalseo-Gu, Daegu, 42601 South Korea; 2 Department of Occupational and Environmental Medicine, Ewha Womans University School of Medicine, 911-1 Mok-6-dong, Yangchun-gu, Seoul, 158-710 South Korea; 3 Institute of Health Policy and Management, Seoul National University Medical Research Center, 103 Daehak-ro, Jongno-gu, Seoul, 110-799 South Korea

**Keywords:** Breast cancer, Socioeconomic inequality, Age-period-cohort effect, Korea

## Abstract

**Background:**

A steadily increasing pattern of breast cancer mortality has been reported in South Korea since the late 1980s. This paper explored the trends of educational inequalities of female breast cancer mortality between 1983 and 2012 in Korea, and conducted age-period-cohort (APC) analysis by educational level.

**Methods:**

Age-standardized mortality rates of breast cancer per 100,000 person-years were calculated. Relative index of inequality (RII) for breast cancer mortality was used as an inequality measure. APC analyses were conducted using the Web tool for APC analysis provided by the Division of Cancer Epidemiology and Genetics at the U.S. National Cancer Institute.

**Results:**

An increasing trend in breast cancer mortality among Korean women between 1983 and 2012 was due to the increased mortality of the lower education groups (i.e., no formal education or primary education and secondary education groups), not the highest education group. The breast cancer mortality was higher in women with a tertiary education than in women with no education or a primary education during 1983-1992, and the reverse was true in 1993-2012. Consequently, RII was changed from positive to negative associations in the early 2000s. The lower education groups had the increased breast cancer mortality and significant cohort and period effects between 1983 and 2012, whereas the highest group did not.

**Conclusions:**

APC analysis by socioeconomic position used in this study could provide an important clue for the causes on breast cancer mortality. The long-term monitoring of socioeconomic patterning in breast cancer risk factors is urgently needed.

**Electronic supplementary material:**

The online version of this article (doi:10.1186/s12939-017-0554-6) contains supplementary material, which is available to authorized users.

## Background

Breast cancer was the leading cause of cancer death and the most commonly diagnosed cancer among women worldwide in 2012 [1]. In South Korea (hereafter ‘Korea’), breast cancer was the second most diagnosed cancer following thyroid cancer, and the sixth fatal cancer for women in 2012 [2]. While decreasing patterns of breast cancer mortality rates were found in western countries since the 1990s, a steadily increasing pattern of breast cancer mortality has been reported in Korea since the late 1980s [3].

Breast cancer was viewed as a “cancer of affluence” because the incidence and mortality of breast cancer was positively related to high socioeconomic position and more developed countries [4, 5]. Although the positive relationship between breast cancer mortality and socioeconomic position has still been observed frequently [6], breast cancer mortality among the low socioeconomic groups has been catching up with the high socioeconomic groups [7]. Several recent studies reported negative or no association between breast cancer mortality and socioeconomic position [8–11]. Among those, the change of inequality direction on breast cancer mortality in Korea was most prominent. Educational inequalities of breast cancer mortality were changed from a significant positive association in 2001 to a significant negative association in 2011, and this trend was first observed in younger women [9, 12].

Age-period-cohort (APC) effect analysis can provide clues of the cause of the disease through describing temporal variations of disease incidence or mortality. APC effect on breast cancer has been analyzed in many studies including those done in Korea [13–16]. However, to the best of our knowledge, there has been no study analyzing APC effect considering socioeconomic position. Especially in the situation where each socioeconomic position group has different trends like breast cancer mortality in Korea, APC effect analysis might supply more salient information.

In this study we examined the trends of educational inequalities of female breast cancer mortality between 1983 and 2012 in Korea, and conducted APC analysis by educational level.

## Methods

### Data

Female breast cancer (C500-C509 in International Classification of Disease-10) mortality data (death certificate data) from 1983 to 2012 and the National Census data in 1985, 1990, 1995, 2000, 2005, and 2010 were obtained from Micro Data Service System provided by Statistics Korea (http://mdis.kostat.go.kr/). Female breast cancer mortality data were categorized into seven 5-year age groups (25-29, 30-34, 35-39, … 55-59), six 5-year calendar periods (1983-1987, 1988-1992, 1993-1997, 1998-2002, 2003-2007, 2008-2012), and three educational levels (no formal education or primary: 0-6 years, secondary: 7-12 years, tertiary: 13+). The National Census data were used as the denominators (numbers of women at risk) and categorized into seven 5-year age groups and three educational levels for each census year. The 5-year birth cohort groups were defined by subtracting age from period based on the midpoint of the five-year age band and the mid-year of the five-year calendar period.

### Statistical analysis

Age-standardized mortality rates of breast cancer per 100,000 person-years for each 5-year calendar period were calculated by the direct method with the 2005 Korean census population as the standard population. The breast cancer mortality rate ratio and 95% confidence interval (95% CI) for each 5-year calendar period was estimated using Poisson regression and adjusted for age. Relative index of inequality (RII) for breast cancer mortality was used as an inequality measure. To obtain RII, we ordered the educational groups from highest (tertiary) to lowest (no formal education or primary) and calculated the cumulative percentage distribution of each educational group by 5-year age groups and 5-year calendar periods. Poisson regression model was used to estimate the RII. These analyses were performed using SAS software version 9.3 (SAS Institute, Cary, NC, USA).

APC analyses were conducted using the Web tool for APC analysis provided by Division of Cancer Epidemiology and Genetics at the U.S. National Cancer Institute (http://analysistools.nci.nih.gov/apc/). Additional details of the Web tool are described elsewhere [17]. From the Web tool, we obtained estimated annual percentage changes of the age-standardized rates (net drift), estimated age-specific annual percentage change over time (local drifts), ratio of longitudinal versus cross-sectional age curves, ratio of age-specific rates in each period relative to reference period 1993-1997 (period rate ratios), and ratio of age-specific rates in each cohort relative to reference cohort 1953 (cohort rate ratios). In all APC analyses, the reference groups were the central age group, central calendar period, and central birth cohort group. We created all figures using the R statistical programming language. Additionally, total number of female breast cancer deaths and person-years according to age groups, and age-specific mortality rate by educational level during1983-2012 were presented in Additional file 1: Table S1 and Additional file 2: Table S2.

## Results

**Fig. 1:**
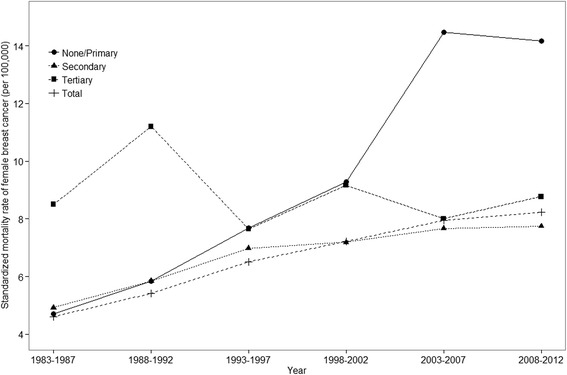
Age-standardized (reference population = 2005 Korean census) mortality rate of breast cancer by educational level between 1983 and 2012 among Korean women

Figure 1 shows the age-standardized mortality rate of female breast cancer per 100,000 person-years according to educational level between 1983 and 2012 in Korea. The age-standardized mortality rate of breast cancer for all Korean women increased during the last three decades from 4.61 (95% CI: 4.39-4.83) to 8.22 (95% CI: 8.00-8.43). Women with no formal education or a primary education and with a secondary education showed an increasing age-standardized mortality rate during study period, whereas women with a tertiary education had no consistent trend. Women with a tertiary education showed the highest mortality rate between 1983-1987 and 1988-1992, and then lower than women with no formal education or primary education (see Additional file 3: Table S3 for the detail numbers).

Table 1 presents the age-adjusted mortality rate ratio and RII for breast cancer mortality according to 5 year calendar periods. The risk of breast cancer death among women with no formal education or primary education was 0.66 (95% CI: 0.54-0.82) times compared to women with a tertiary education in 1983-1987, and that increased to 1.31 (95% CI: 1.19-1.43) in 2008-2012. The RII for breast cancer mortality showed a significant increased trend across the full period (*p* value for trend < 0.0001). The estimated RII for breast cancer mortality comparing the lowest with the highest education categories was 0.72 (95% CI = 0.60 to 0.85) in 1988-1992 and 1.23 (95% CI = 1.10 to 1.36) in 2008-2012 (Table 1).

**Table 1:** Age-adjusted mortality rate ratio and relative index of inequality for breast cancer mortality according to 5 year calendar periods

	Rate ratio			RII
	None/Primary	Secondary	Tertiary	
1983-1987	0.66 (0.54-0.82)	0.67 (0.54-0.82)	1 (reference)	0.84 (0.68-1.03)
1988-1992	0.60 (0.51-0.70)	0.63 (0.54-0.73)	1 (reference)	0.72 (0.60-0.85)
1993-1997	0.96 (0.84-1.10)	1.03 (0.91-1.16)	1 (reference)	0.92 (0.80-1.07)
1998-2002	0.86 (0.77-0.96)	0.87 (0.79-0.95)	1 (reference)	0.88 (0.77-1.00)
2003-2007	1.38 (1.25-1.52)	1.05 (0.97-1.13)	1 (reference)	1.45 (1.29-1.63)
2008-2012	1.31 (1.19-1.43)	0.91 (0.86-0.97)	1 (reference)	1.23 (1.10-1.36)
*p* value for trend				<.0001

Table 2 shows the estimated annual percentage changes of the age-standardized breast cancer mortality rates and the estimated age-specific annual percentage change over time by educational level. Breast cancer mortality rates increased 3.85% annually among women with no formal education or primary education, while among women with a tertiary education it showed no significant annual change. Age-specific annual percentage change in the breast cancer mortality rates during the study periods showed significant positive value among women with no formal education or primary education and women with a secondary education aged between 30-34 and 55-59. No significant age-specific annual percentage change occurred among women with a tertiary education (Table 2).

**Table 2:** Estimated annual percentage changes (95% CI) of the age-standardized breast cancer mortality rates and estimated age-specific annual percentage change (95% CI) over time

		Educational level		
	Total	None or primary	Secondary	Tertiary
Net drift (95% CI)	1.87 (1.63-2.11)	3.85 (1.23-6.53)	1.89 (1.51-2.27)	-0.34 (-1.11-0.43)
*p*-value	<0.001	0.004	<0.001	0.386
Local drifts (95% CI)				
25-29	-0.40 (-1.35-0.55)	-4.45 (-20.42-14.72)	1.35 (-0.19-2.90)	-0.29 (-1.93-1.39)
30-34	0.67 (0.18-1.16)	4.29 (0.80-7.90)	2.08 (1.36-2.81)	-0.14 (-1.26-1.00)
35-39	1.79 (1.46-2.12)	6.13 (4.68-7.60)	2.80 (2.34-3.26)	0.40 (-0.59-1.40)
40-44	1.82 (1.54-2.09)	5.75 (4.97-6.54)	1.92 (1.53-2.32)	0.09 (-0.85-1.04)
45-49	2.28 (2.03-2.54)	5.22 (4.76-5.68)	1.43 (1.04-1.82)	-0.36 (-1.36-0.65)
50-54	3.02 (2.72-3.31)	4.83 (4.43-5.24)	1.29 (0.77-1.81)	-1.14 (-2.46-0.20)
55-59	3.87 (3.41-4.33)	4.44 (3.89-4.99)	2.23 (1.16-3.31)	-0.99 (-3.43-1.51)
*p*-value	<0.001	0.001	<0.001	0.272

Net drift: Estimated annual percentage changes of the age-standardized rates

Local drifts: Estimated age-specific annual percentage change over time

**Fig. 2:**
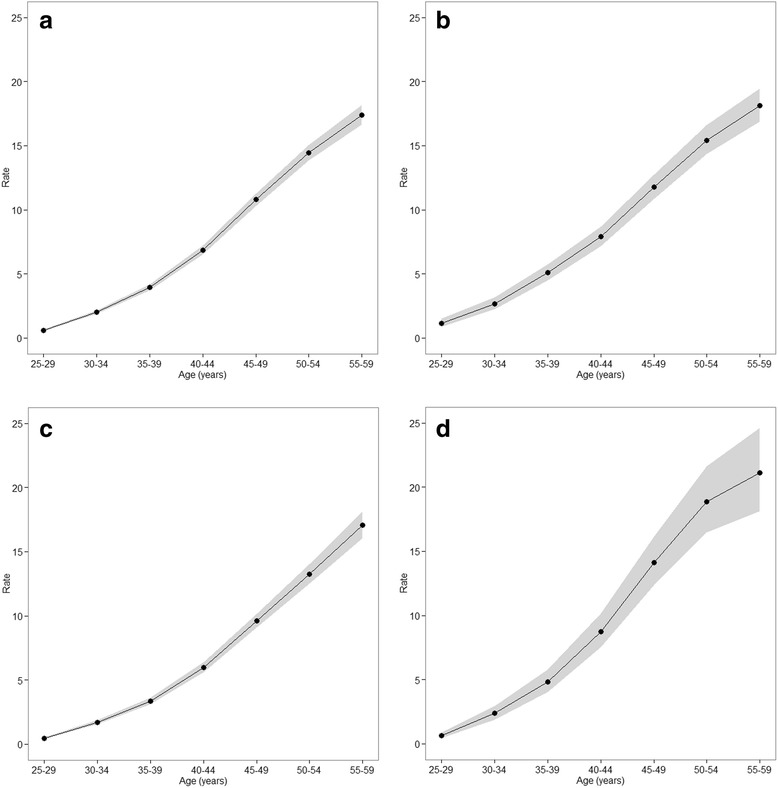
Longitudinal age curves of female breast cancer mortality by educational level; Expected age-specific rates in reference cohort c0 adjusted for period effects **a** total, **b** none or primary, **c** secondary, **d** tertiary

Figure 2 presents the longitudinal age curves of female breast cancer mortality by educational level. The risks of breast cancer death increased in all education groups. The secondary education group (Fig. 2c) shows the lowest rate at all age groups, while the tertiary education group (Fig. 2d) shows the highest rate at ages 40-44 and thereafter (Fig. 2).

**Fig. 3:**
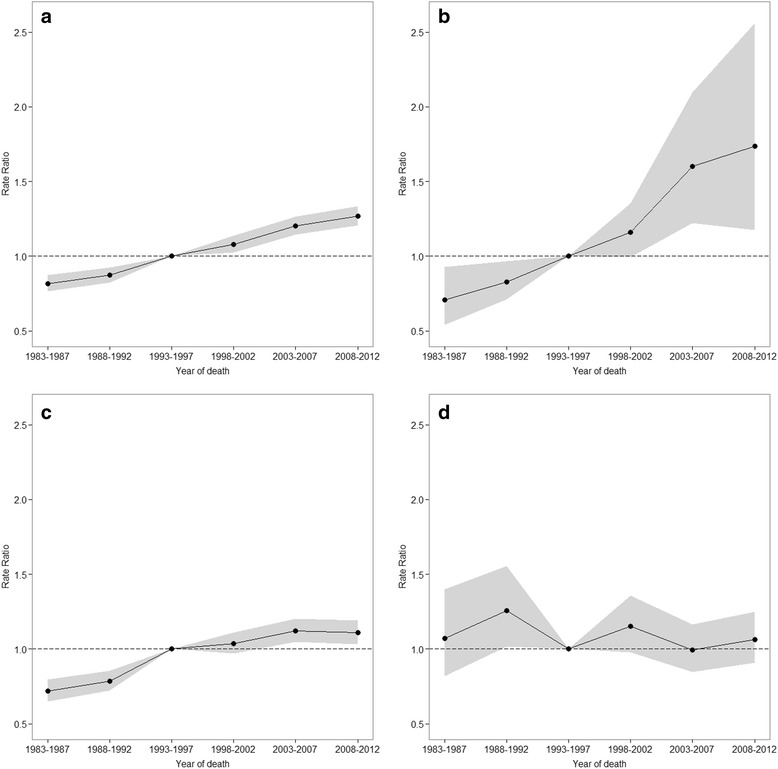
Period rate ratios by educational level: the period relative risk adjusted for age and non-linear cohort effects in each period relative to reference period 1993-1997; **a** total, **b** none or primary, **c** secondary, **d** tertiary

Figure 3 shows the estimated period effects by educational level. The no formal education or primary education group (Fig. 3b) shows an upward pattern during the study periods, while the tertiary education group (Fig. 3d) shows no significant increase except 1988-1992 (Fig. 3). Additional file 4: Table S4 shows Wald tests results for period effects. Period effects were statistically significant for the no formal education or primary education group (*p* = 0.002) and the secondary education group (*p* < 0.0001) (Additional file 4: Table S4).

**Fig. 4:**
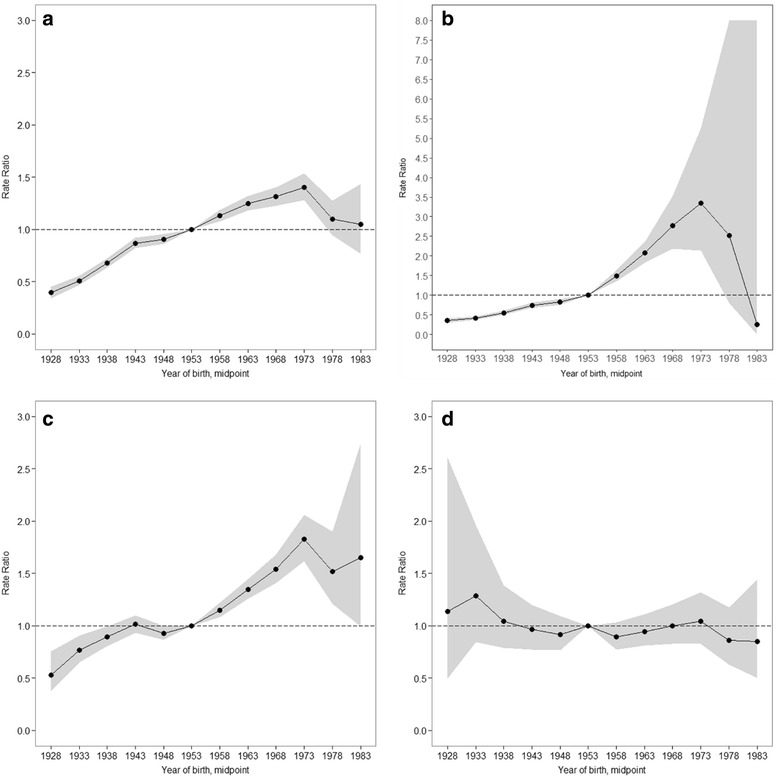
Cohort rate ratios by educational level: the cohort relative risk adjusted for age and non-linear period effects in each cohort relative to reference cohort 1953; **a** total, **b** none or primary, **c** secondary, **d** tertiary

Figure 4 shows the estimated cohort effects by educational level. Women from the 1928 birth cohort to the 1973 birth cohort had increasing cohort effects on breast cancer mortality over time, especially among the no formal education or primary education group (Fig. 4b) and the secondary education group (Fig. 4c), while the tertiary education group (Fig. 4d) showed no significant increase (Fig. 4). Additional file 4: Table S4 shows Wald tests results for cohort effects. Cohort effects were statistically significant for the no formal education or primary education group (*p* < 0.0001) and the secondary education group (*p* < 0.0001) (Additional file 4: Table S4).

## Discussion

This study showed that an increasing trend in breast cancer mortality among Korean women between 1983 and 2012 was due to the increased mortality of the lower education groups (i.e., no formal education or primary education and secondary education groups), not the highest education group. The breast cancer mortality was higher in women with a tertiary education than in women with no education or a primary education during 1983-1992, and the reverse was true in 1993-2012. Consequently, RII was changed from positive to negative associations in the early 2000s. We also found significant cohort and period effects in the lower education groups, while cohort and period effects were not observed in the highest education group.

Breast cancer has been one of the well-known diseases with a positive social gradient [6, 18, 19]. Although no significant associations were observed in some countries [11, 20], most western countries have a consistent positive association by education in breast cancer mortality [6]. This study showed educational inequality for breast cancer mortality before the early 2000s in Korea also had a positive association like most western countries. However, after the early 2000s, the RII for breast cancer mortality was changed to a negative association, which was mainly related to the rapid increase in breast cancer mortality of the lowest education group for the last 30 years.

The change of the inequality pattern could be explained by the changes of the incidence, survival, and risk factors across education groups. Breast cancer incidence among Korean women increased during 1993-2012 [2, 21]. However, the changing patterns between the incidence of breast cancer and socioeconomic position were observed during these periods. According to Korean hospital-based studies, women with a low educational level had a significantly lower incidence of breast cancer in 1994 [22], while women with a high educational level had a significantly lower incidence in 2004 [23]. In addition, a national level study showed that breast cancer incidence inequality which was presented as RII by household income was not significant in 2001 [24]. Based on these studies, we can assume that the incidence of breast cancer might be changed from less favorable to more favorable for the higher education group during the last decades, which could be one factor that led the change of breast cancer mortality inequality by educational level.

Furthermore, socioeconomic breast cancer mortality disparities could be partially explained by the risk factors related to the survival rate such as screening, tumor stage distribution at diagnosis, and treatment inequalities [25]. The National Cancer Screening Program for breast cancer was started in 1999 in Korea [26]. This program provided free cancer screening for the medical aid beneficiary group at first, and extended gradually to the national health insurance beneficiaries in the bottom 50% of income in 2006. Despite this program, women with a low socioeconomic position were less likely to attend the screening program [27, 28], and the trend of inequality in screening attendance did not decrease between 1998 and 2012 [29]. Thus, women with a low socioeconomic position were more likely to have had a delayed diagnosis and significantly worse tumor stage [30, 31]. After diagnosis, women with a low socioeconomic position were less likely to have had optimal treatment [32], and thus less likely to survive [33]. Nonetheless, it should be noted that inequality of survival factors could contribute to the increase of breast cancer mortality inequality, but that could not explain the increase of breast cancer mortality itself in the lower education group because survival rates from breast cancer in Korea have increased consistently [2, 34].

In addition, several risk factors might have contributed to the mortality disadvantages among the less educated. For example, the prevalence of obesity in Korea was higher in the lower education group and the inequality for abdominal obesity by educational group increased between 1998 and 2007 [35]. The rate of oral contraceptive use of the lower education group was higher than that of the highest education group in 2005 [36]. Those factors could have attributed to the increase of breast cancer mortality in the lower education group. However, considering the incubation period from the exposure to the risk factors to the occurrence of cancer, those results with a limited time period seemed to lack the necessary evidence to explain the unique increase of breast cancer mortality in the lower education group.

Analysis results showed that the lower education groups had the increased breast cancer mortality and significant cohort and period effects between 1983 and 2012, whereas the highest group did not. The risk factors which could accompany the cohort effects in breast cancer incidence are reproductive factors (e.g., early menarche, late menopause, delayed age at first birth, and less breastfeeding) and behavioural factors (e.g., high fat diet, obesity, alcohol use, and use of exogenous hormone) [37]. Behavioural factors could be related to the predominant lifestyle and food and drug supply of the time period, and could produce the period effect as well. Most above risk factors were suggested as explanations for the increasing cohort effects on breast cancer mortality in the previous studies, because the prevalence of the risk factors also has increased with economic development and westernization of lifestyle in Korea [14, 37].

This study also showed that women with a lower education had higher breast cancer death rates in more current cohorts in contrast to the highest education group. It means that if the risk factors with the different pattern of the prevalence by educational level were found, it would be reasonable that those factors were considered as main risk factors for the increase of breast cancer mortality. However, unfortunately, there were few studies that contained evidence to conclude what the main risk factors were. Reproductive factors including age at first birth were still less favorable for the higher education group in Korea [38], likewise with western countries [39, 40], and the study which supported the decreasing gap in reproductive factors between educational groups could not be found. The gap of age at first birth by educational level among the 1960-1964 birth cohort, the 1965-1969 birth cohort, and the 1970-1974 birth cohort was not different [41], although the gap of the number of live births by educational level decreased between 1980 and 2000 [42].

This study has limitations. First, this study is ecological, thereby we can only speculate on the etiologies of the observed changes. Second, population distribution of educational attainment among Korean women changed dramatically during 1983 to 2012. Only 6.2% of women attained a tertiary education during 1983-1987. The percentage increased to 38.9% in 2008-2012, while women with no or a primary education only accounted for 7.2% in 2008-2012 (see Additional file 1: Table S1 for the population distribution of educational attainment). Large standard errors, which should be interpreted with caution, resulted from the fact that the highest education group in the 1980s and the lowest education group in the 2000s had the small number of deaths, despite our having used figures for total population and deaths.

On the other hand, this study has an important strength in that, to the best of our knowledge, this is the first study to analyze the APC effects on female breast cancer mortality by educational level over 30 years. APC analysis by socioeconomic position could be applied to explore the causes of other diseases with epidemiologic transition.

## Conclusions

In conclusion, this study showed that the lower education groups had the increased breast cancer mortality and significant cohort and period effects between 1983 and 2012, whereas the highest group did not. This approach could provide an important clue for the cause on the trend of breast cancer mortality. Considering the increasing mortality of breast cancer and the widening gap in breast cancer mortality between the high and low education groups, the long-term monitoring of socioeconomic patterning in breast cancer risk factors are urgently needed.

## Additional files

Additional file 1: Table S1. Total number of female breast cancer death and person-years according to age groups, and % person years for educational groups during1983-2012 in Korea. (DOCX 14 kb)

Additional file 2: Table S2. Age-specific mortality rate per 100,000 person-years during1983-2012 in Korea. (DOCX 14 kb)

Additional file 3: Table S3. Age-standardized (reference population = 2005 census) mortality rate of breast cancer by educational level among Korean women according to 5 year calendar periods between 1983 and 2012. (DOCX 13 kb)

Additional file 4: Table S4. Rate ratios (95% CI) of periods and cohorts by educational level. (DOCX 14 kb)

## Abbreviations

APC: Age-period-cohort
RII: Relative index of inequality
