# Supplementary material for: Increased breast cancer mortality only in the lower education group: age-period-cohort effect in breast cancer mortality by educational level in South Korea, 1983-2012
Source: Int J Equity Health. 2017 Mar 31;16:56. doi: 10.1186/s12939-017-0554-6 (PMC5374568; doi:10.1186/s12939-017-0554-6)
Supplement: Supplementary file 1 — Total number of female breast cancer death and person-years according to age groups, and % person years for educational groups during1983-2012 in Korea. (DOCX 14 kb) [file 12939_2017_554_MOESM1_ESM.docx]

Table S1. Total number of female breast cancer death and person-years according to age groups, and % person years for educational groups during1983-2012 in Korea.

|  | Year of death | | | | | |
| --- | --- | --- | --- | --- | --- | --- |
| Age group | 1983-1987 | 1988-1992 | 1993-1997 | 1998-2002 | 2003-2007 | 2008-2012 |
|  | Number of deaths | | | | | |
| 25-29 | 74 | 73 | 91 | 82 | 45 | 52 |
| 30-34 | 141 | 217 | 283 | 262 | 294 | 210 |
| 35-39 | 218 | 271 | 476 | 526 | 548 | 556 |
| 40-44 | 340 | 370 | 536 | 738 | 898 | 897 |
| 45-49 | 385 | 516 | 588 | 813 | 1195 | 1306 |
| 50-54 | 320 | 497 | 628 | 769 | 1042 | 1555 |
| 55-59 | 243 | 379 | 579 | 753 | 937 | 1207 |
|  | Person-years | | | | | |
| 25-29 | 10215110 | 10862385 | 10295620 | 10197060 | 9067575 | 8680720 |
| 30-34 | 7627235 | 10323845 | 10417120 | 10122925 | 10181845 | 9144755 |
| 35-39 | 6283325 | 7764575 | 10151615 | 10344630 | 10235585 | 10194570 |
| 40-44 | 5393630 | 6119990 | 7454410 | 9831660 | 10203070 | 10299960 |
| 45-49 | 5230740 | 5379010 | 6012515 | 7277315 | 9695200 | 10143585 |
| 50-54 | 4427925 | 5077275 | 5173170 | 5823175 | 7143500 | 9550790 |
| 55-59 | 3535625 | 4309100 | 4948330 | 5042305 | 5757205 | 7029740 |
| Education | % person years | | | | | |
| None/Primary | 45.7 | 32.7 | 22.4 | 16.1 | 10.2 | 7.2 |
| Secondary | 48.1 | 57.4 | 61.6 | 61.9 | 58.1 | 53.9 |
| Tertiary | 6.2 | 9.9 | 15.9 | 22 | 31.7 | 38.9 |
