# Supplementary material for: Increased breast cancer mortality only in the lower education group: age-period-cohort effect in breast cancer mortality by educational level in South Korea, 1983-2012
Source: Int J Equity Health. 2017 Mar 31;16:56. doi: 10.1186/s12939-017-0554-6 (PMC5374568; doi:10.1186/s12939-017-0554-6)
Supplement: Supplementary file 2 — Age-specific mortality rate per 100,000 person-years during1983-2012 in Korea. (DOCX 14 kb) [file 12939_2017_554_MOESM2_ESM.docx]

Table S2. Age-specific mortality rate per 100,000 person-years during1983-2012 in Korea.

|  | Year of death | | | | | |
| --- | --- | --- | --- | --- | --- | --- |
| Age group | 1983-1987 | 1988-1992 | 1993-1997 | 1998-2002 | 2003-2007 | 2008-2012 |
|  | Total | | | | | |
| 25-29 | 0.72 | 0.67 | 0.88 | 0.80 | 0.50 | 0.60 |
| 30-34 | 1.85 | 2.10 | 2.72 | 2.59 | 2.89 | 2.30 |
| 25-39 | 3.47 | 3.49 | 4.69 | 5.08 | 5.35 | 5.45 |
| 40-44 | 6.30 | 6.05 | 7.19 | 7.51 | 8.80 | 8.71 |
| 45-49 | 7.36 | 9.59 | 9.78 | 11.17 | 12.33 | 12.88 |
| 50-54 | 7.23 | 9.79 | 12.14 | 13.21 | 14.59 | 16.28 |
| 55-59 | 6.87 | 8.80 | 11.70 | 14.93 | 16.28 | 17.17 |
|  | No education/Primary | | | | | |
| 25-29 | 1.63 | 2.69 | 1.89 | 4.11 | 0.00 | 0.00 |
| 30-34 | 2.66 | 3.42 | 6.58 | 6.95 | 12.51 | 7.12 |
| 35-39 | 3.90 | 4.51 | 8.93 | 9.55 | 17.95 | 13.01 |
| 40-44 | 5.98 | 6.85 | 7.74 | 9.70 | 19.71 | 19.90 |
| 45-49 | 6.66 | 8.92 | 9.59 | 11.57 | 18.82 | 21.25 |
| 50-54 | 6.84 | 8.55 | 10.63 | 12.16 | 16.28 | 22.93 |
| 55-59 | 6.46 | 7.14 | 9.79 | 12.97 | 15.95 | 18.22 |
|  | Secondary | | | | | |
| 25-29 | 0.51 | 0.48 | 1.03 | 0.77 | 0.64 | 0.72 |
| 30-34 | 1.43 | 1.80 | 2.74 | 2.59 | 3.28 | 2.48 |
| 35-39 | 3.13 | 3.09 | 4.14 | 4.66 | 5.52 | 5.73 |
| 40-44 | 6.44 | 5.17 | 6.63 | 7.26 | 8.38 | 8.17 |
| 45-49 | 8.36 | 9.61 | 9.60 | 10.31 | 11.27 | 11.89 |
| 50-54 | 8.27 | 11.79 | 14.38 | 13.25 | 13.37 | 14.26 |
| 55-59 | 8.66 | 13.95 | 16.19 | 17.36 | 16.18 | 16.08 |
|  | Tertiary | | | | | |
| 25-29 | 0.77 | 0.72 | 0.51 | 0.79 | 0.41 | 0.55 |
| 30-34 | 2.33 | 2.48 | 1.85 | 2.38 | 2.35 | 2.16 |
| 35-39 | 3.62 | 3.68 | 4.12 | 5.36 | 4.58 | 5.04 |
| 40-44 | 9.04 | 8.56 | 9.67 | 6.73 | 7.83 | 9.05 |
| 45-49 | 14.48 | 17.60 | 11.96 | 15.81 | 11.73 | 13.26 |
| 50-54 | 14.09 | 21.94 | 11.69 | 18.89 | 17.57 | 18.32 |
| 55-59 | 22.70 | 37.65 | 19.90 | 22.53 | 18.64 | 20.43 |
