# Supplementary material for: Increased breast cancer mortality only in the lower education group: age-period-cohort effect in breast cancer mortality by educational level in South Korea, 1983-2012
Source: Int J Equity Health. 2017 Mar 31;16:56. doi: 10.1186/s12939-017-0554-6 (PMC5374568; doi:10.1186/s12939-017-0554-6)
Supplement: Supplementary file 3 — Age-standardized (reference population = 2005 census) mortality rate of breast cancer by educational level among Korean women according to 5 year calendar periods between 1983 and 2012. (DOCX 13 kb) [file 12939_2017_554_MOESM3_ESM.docx]

Table S3. Age-standardized (reference population=2005 census) mortality rate of breast cancer by educational level among Korean women according to 5 year calendar periods between 1983 and 2012.

|  | Total | None/Primary | Secondary | Tertiary |
| --- | --- | --- | --- | --- |
| 1983-1987 | 4.61 (4.39-4.83) | 4.71 (4.40-5.01) | 4.92 (4.45-5.38) | 8.50 (6.39-10.62) |
| 1988-1992 | 5.42 (5.20-5.65) | 5.84 (5.44-6.24) | 5.84 (5.43-6.25) | 11.20 (9.28-13.11) |
| 1993-1997 | 6.50 (6.27-6.73) | 7.69 (7.04-8.35) | 6.98 (6.62-7.34) | 7.65 (6.61-8.70) |
| 1998-2002 | 7.22 (6.99-7.44) | 9.28 (8.15-10.40) | 7.19 (6.88-7.51) | 9.16 (8.26-10.07) |
| 2003-2007 | 7.94 (7.72-8.16) | 14.48 (12.52-16.44) | 7.67 (7.39-7.95) | 8.01 (7.41-8.61) |
| 2008-2012 | 8.22 (8.00-8.43) | 14.16 (11.94-16.38) | 7.74 (7.47-8.01) | 8.77 (8.27-9.26) |
