# Supplementary material for: Increased breast cancer mortality only in the lower education group: age-period-cohort effect in breast cancer mortality by educational level in South Korea, 1983-2012
Source: Int J Equity Health. 2017 Mar 31;16:56. doi: 10.1186/s12939-017-0554-6 (PMC5374568; doi:10.1186/s12939-017-0554-6)
Supplement: Supplementary file 4 — Rate ratios (95% CI) of periods and cohorts by educational level. (DOCX 14 kb) [file 12939_2017_554_MOESM4_ESM.docx]

Table S4. Rate ratios (95% CI) of periods and cohorts by educational level.

|  |  | Educational level | | |
| --- | --- | --- | --- | --- |
|  | Total | None/Primary | Secondary | Tertiary |
| Year of death, midpoint | |  |  |  |
| 1985 | 0.82 (0.77-0.88) | 0.71 (0.54-0.93) | 0.72 (0.65-0.80) | 1.07 (0.82-1.40) |
| 1990 | 0.87 (0.82-0.93) | 0.83 (0.71-0.97) | 0.79 (0.72-0.86) | 1.26 (1.02-1.56) |
| 1995 | 1.00 | 1.00 | 1.00 | 1.00 |
| 2000 | 1.08 (1.03-1.14) | 1.16 (0.99-1.35) | 1.04 (0.97-1.11) | 1.15 (0.98-1.36) |
| 2005 | 1.20 (1.14-1.27) | 1.60 (1.22-2.10) | 1.12 (1.05-1.20) | 0.99 (0.85-1.16) |
| 2010 | 1.27 (1.21-1.34) | 1.74 (1.18-2.56) | 1.11 (1.03-1.19) | 1.07 (0.91-1.25) |
| p-value | <0.0001 | 0.002 | <0.0001 | 0.066 |
| Year of birth, midpoint | |  |  |  |
| 1928 | 0.39 (0.34-0.45) | 0.35 (0.30-0.41) | 0.53 (0.37-0.76) | 1.14 (0.50-2.61) |
| 1933 | 0.51 (0.47-0.56) | 0.42 (0.37-0.47) | 0.77 (0.65-0.91) | 1.29 (0.85-1.96) |
| 1938 | 0.68 (0.63-0.72) | 0.55 (0.51-0.61) | 0.90 (0.81-0.99) | 1.05 (0.79-1.39) |
| 1943 | 0.87 (0.82-0.92) | 0.74 (0.68-0.81) | 1.01 (0.94-1.10) | 0.96 (0.77-1.20) |
| 1948 | 0.91 (0.86-0.95) | 0.82 (0.76-0.90) | 0.93 (0.87-1.00) | 0.92 (0.77-1.09) |
| 1953 | 1.00 | 1.00 | 1.00 | 1.00 |
| 1958 | 1.13 (1.08-1.19) | 1.49 (1.36-1.63) | 1.15 (1.08-1.22) | 0.89 (0.77-1.03) |
| 1963 | 1.25 (1.18-1.32) | 2.08 (1.83-2.36) | 1.35 (1.26-1.45) | 0.95 (0.81-1.11) |
| 1968 | 1.31 (1.23-1.40) | 2.77 (2.19-3.52) | 1.54 (1.41-1.68) | 1.00 (0.83-1.20) |
| 1973 | 1.40 (1.28-1.54) | 3.35 (2.13-5.26) | 1.83 (1.62-2.06) | 1.05 (0.83-1.32) |
| 1978 | 1.10 (0.94-1.28) | 2.52 (0.80-8.00) | 1.52 (1.21-1.90) | 0.86 (0.63-1.18) |
| 1983 | 1.05 (0.76-1.43) | 0.26 (0.00-150.24) | 1.65 (0.99-2.75) | 0.85 (0.50-1.44) |
| p-value | <0.0001 | <0.0001 | <0.0001 | 0.594 |
